# Integrated Physiological, Transcriptomic, and Proteomic Analyses Reveal the Regulatory Role of Melatonin in Tomato Plants’ Response to Low Night Temperature

**DOI:** 10.3390/antiox11102060

**Published:** 2022-10-19

**Authors:** Xiaolong Yang, Yumeng Zhang, Ting Liu, Jiali Shi, Mingfang Qi, Yufeng Liu, Tianlai Li

**Affiliations:** 1 Key Laboratory of Protected Horticulture of Ministry of Education, National & Local Joint Engineering Research Center of Northern Horticultural Facilities Design & Application Technology (Liaoning), College of Horticulture, Shenyang Agricultural University, Shenyang 110866, China; 2 College of Horticulture, South China Agricultural University, Guangzhou 510642, China; 3 Jiuquan Academy of Agricultural Sciences, Jiuquan 735099, China

**Keywords:** melatonin, antioxidant, tomato, abiotic stress, photosynthesis

## Abstract

Melatonin is a direct free radical scavenger that has been demonstrated to increase plants’ resistance to a variety of stressors. Here, we sought to examine the effect of melatonin on tomato seedlings subjected to low night temperatures using an integrated physiological, transcriptomic, and proteomic approach. We found that a pretreatment with 100 μM melatonin increased photosynthetic and transpiration rates, stomatal apertures, and peroxidase activity, and reduced chloroplast damage of the tomato plant under a low night temperature. The melatonin pretreatment reduced the photoinhibition of photosystem I by regulating the balance of both donor- and acceptor-side restriction of PSI and by increasing electron transport. Furthermore, the melatonin pretreatment improved the photosynthetic performance of *proton gradient regulation 5* (*SlPGR5*) and *SlPGR5-like photosynthetic phenotype 1* (*SlPGRL1*)-suppressed transformants under a low night temperature stress. Transcriptomic and proteomic analyses found that the melatonin pretreatment resulted in the upregulation of genes and proteins related to transcription factors, signal transduction, environmental adaptation, and chloroplast integrity maintenance in low night temperature-stressed tomato plants. Collectively, our results suggest that melatonin can effectively improve the photosynthetic efficiency of tomato plants under a low night temperature and provide novel insights into the molecular mechanism of melatonin-mediated abiotic stress resistance.

## 1. Introduction

As a multifunctional biomolecule, melatonin is capable of directly scavenging free radicals, reactive oxygen species (ROS), and reactive nitrogen species (RNS) in both animals and plants [1]. Melatonin and its intermediates scavenge free radicals through a multi-step reaction cascade, resulting in up to 10 free radicals scavenged per melatonin molecule. In fact, the radical-scavenging capability of melatonin far exceeds that of other more well-known antioxidants, including vitamins C and E, as these are capable of scavenging only one free radical per molecule [1,2]. Melatonin was first discovered in plants in 1995, resulting in a wealth of research into its diverse physiological functions and associated biosynthetic, metabolic, and signal transduction pathways [3,4].

The primary enzymes involved in melatonin biosynthesis and its associated metabolic pathways have been most fully characterized in Arabidopsis and rice [5,6]. In plants, melatonin synthesis begins with the amino acid tryptophan, and the pathway involves at least six enzymes catalyzing four enzymatic reactions [7,8,9,10,11]. Melatonin biosynthesis occurs primarily in the cytoplasm, mitochondria, and chloroplasts, perhaps contributing to the diversity of the physiological functions exhibited by this broad-spectrum antioxidant [5,12]. Melatonin is degraded through two different pathways: non-enzymatic degradation and enzymatic transformation. Occurring in both animals and plants, the non-enzymatic degradation of melatonin involves either reactions with free radicals or other ROS, or photocatalytic reactions upon exposure to UV light [2,13]. In plants, melatonin also undergoes enzymatic transformation, with melatonin-2-hydroxylase (M2H) and melatonin-3-hydroxylase (M3H) catalyzing the formation of 2-hydroxyl melatonin and cyclic 3-hydroxyl melatonin, respectively [14,15].

A considerable amount of recent research has focused on understanding the mechanism by which melatonin protects plants from abiotic stress [16,17,18]. Both the exogenous application of melatonin and the upregulation of endogenous melatonin through genetic modification have been found to enhance plant stress resistance. Across a variety of plants, including apple trees, grapevines, corn, sunflowers, tomato plants, watermelon, and wheat, melatonin has been found to increase antioxidant enzyme activity, alleviate oxidative stress and damage, delay cellular senescence, maintain intracellular redox homeostasis, and alter patterns of gene expression related to hormone metabolism and signal transduction [19,20,21,22,23,24,25,26,27]. Melatonin appears to also be crucial for immune response regulation, lateral root development, flowering phenology, and postharvest shelf-life prolongation. Melatonin is considered a master regulator of plant growth and development through its interactions with plant melatonin receptors (PMTRs), phytohormones, and ROS/NO/Ca^2+^-mediated signal networks [28,29,30,31,32,33]. In addition, research suggests that melatonin can improve chlorophyll synthesis, photosynthetic efficiency, chloroplast integrity, and the accumulation of assimilates [34,35]. Taken together, it seems possible that melatonin may be capable of enhancing the photosynthetic capacity of plants under abiotic stress.

In northern China and other northern regions, a low night temperature is the primary limiting factor for vegetable production in solar greenhouses. Particularly in the tomato plant, research and observation suggest that low night temperatures decrease photosynthetic efficiency, leading to stress and photoinhibition [36,37]. Based on the above analysis of the function of melatonin, we hypothesized that melatonin can improve the resistance of the tomato plant to low night temperatures by regulating photoprotection and inducing physiological and molecular responses. Here, we used an integrated physiological, transcriptomic, and proteomic approach to ascertain the effect of exogenously applied melatonin on the photosynthetic response of tomato plants under low night temperatures. Specifically, we examined changes in the stomatal aperture, chloroplast integrity, endogenous antioxidant enzyme activity, photosynthetic capacity, and transcriptomic and proteomic dynamics. Additionally, we used RNA interference (RNAi) to generate *proton gradient regulation 5* (*SlPGR5*) and *SlPGR5-like photosynthetic phenotype 1* (*SlPGRL1*)-suppressed transformants in order to characterize the involvement of PGR5-dependent cyclic electron flow (CEF) in the melatonin-mediated regulation of photosynthetic efficiency. These results will contribute to our understanding of how melatonin acts to increase plant resistance to abiotic stress in general, and low night temperatures in particular.

## 2. Materials and Methods

### 2.1. Plant Materials and Experimental Treatments

The tomato cultivar ‘Alisa Craig’ was used for all experiments. Seeds were germinated in 50-well seedling trays and were transferred to 13 cm × 13 cm plastic pots when each seedling had two fully expanded true leaves. Seedlings were watered with approximately 50 mL of water per seedling per day. Experimental treatments were carried out when each seedling had four fully expanded true leaves using a climate-controlled, artificially lit grow room at Shenyang Agricultural University, China. The grow room was maintained at 50% humidity during the day, 80% humidity during the night, and a daytime light intensity of 350 μmol·photons·m^−2^·s^−1^. The experimental treatments were as follows: (CK) tomato leaves were sprayed with water and grown at normal temperature; (LN) tomato leaves were sprayed with water and grown at low temperature; (MT) tomato leaves were sprayed with melatonin and grown at normal temperature; (LN) tomato leaves were sprayed with water and grown under a low night temperature; (MTLN) tomato leaves were sprayed with melatonin and grown under a low night temperature. For exogenous melatonin treatments, 100 μM of melatonin was sprayed onto both the top and bottom surfaces of each tomato plant until runoff occurred. Tomato plants were sprayed twice per day, once in the morning and once in the evening, and approximately 40 mL of solution was sprayed onto each plant each day. Normal temperature was controlled at 25/15 °C (day/night; 12 h/12 h), and low night temperature was controlled at 25/6 °C (day/night; 12 h/12 h); the daytime (08:00–20:00) light intensity was 350 μmol·photons·m^−2^·s^−1^. Physiological, transcriptomic, and proteomic parameters were sampled and measured after 7 days of low night temperature treatment.

Additionally, *SlPGR5*-RNAi- and *SlPGRL1*-RNAi-suppressed transformants’ seedlings were used in this study. Transformed tomato plants with four fully expanded true leaves were treated with melatonin as described above, and low night temperature treatment was carried out after 3 consecutive days of spray treatments. The experimental treatments were as follows: (WT) wild-type tomato plants were sprayed with water; (WT+MT) wild-type tomato plants were sprayed with melatonin; (*SlPGR5*-RNAi) *SlPGR5*-RNAi tomato plants were sprayed with water; (*SlPGR5*-RNAi+MT) *SlPGR5*-RNAi tomato plants were sprayed with melatonin; (*SlPGRL1*-RNAi) *SlPGRL1*-RNAi tomato plants were sprayed with water; (*SlPGRL1*-RNAi+MT) *SlPGRL1*-RNAi tomato plants were sprayed with melatonin. Photosynthetic parameters were measured after 7 days of low night temperature treatment (as described above).

### 2.2. Measurement of Malondialdehyde Content and Antioxidant Enzyme Activities

Approximately 0.5 g of fresh tomato leaves were ground in liquid nitrogen and mixed with 10 mL of phosphate-buffered saline (PBS; pH 7.4). Commercial plant malondialdehyde (MDA) enzyme-linked immunosorbent assay (ELISA) kits (Mmbio, Jiangsu, China) were used to determine the MDA content of tomato leaves. The activity of the endogenous antioxidant enzymes superoxide dismutase (SOD), catalase (CAT), and peroxidase (POD) was determined using commercial ELISA kits (Mmbio, Jiangsu, China). Sample preparation and test procedures were carried out in strict accordance with the manufacturer’s instructors [38].

### 2.3. Observation of Stomatal Aperture

Three to five leaves were randomly sampled from tomato plants from each treatment in order to observe stomatal morphology. Prior to microscopic observation, the abaxial leaf epidermis was removed with tweezers and incubated in distilled water. Stomatal apertures were observed with an Axio Observer A1 inverted fluorescence microscope (Zeiss, Oberkochen, Germany). Approximately six random photos were taken for each leaf.

### 2.4. Observation of Chloroplast Ultrastructure

Fragments of veinless strips (1 mm × 2 mm) of tomato leaves were fixed in 2.5% glutaraldehyde solution. Samples were vacuumed until each leaf fragment was fully submerged at the bottom of the solution, and samples were subsequently rinsed and incubated in 1% Acetic acid (OsO_4_, pH 7.2) overnight at 4 °C. Gradient dehydration was conducted using a series of ethanol concentrations after staining with uranyl acetate. Dehydrated samples were embedded in epoxy resin, thinly sliced with an EM UC7 ultramicrotome (Leica, Wetzlar, Germany). Chloroplast ultramicrostructure was observed and photographed using an HT-7700 transmission electron microscope (TEM) (Hitachi, Ibaraki, Japan) [38].

### 2.5. Measurement of Photosynthetic Gas Exchange

The GFS-3000 gas-exchange system and Dual-PAM-100 fluorescence-measuring system (Heinz Walz, Effeltrich, Germany) were used in this study. A 10-L air buffer bottle was connected to the gas-exchange system during measurement, using atmospheric carbon dioxide (CO_2_) content as a reference. The room temperature was approximately 25 °C and the tested light intensity was 1100 μmol·photos·m^−2^·s^−1^. Gas exchange parameters, including net photosynthetic rate (Pn), stomatal conductance (GH_2_O), intercellular CO_2_ concentration (Ci), and transpiration rate (E), were recorded after photosynthesis reached a stable state.

### 2.6. Measurement of the Photochemical Efficiency of PSI and PSII

Chlorophyll (Chl) fluorescence and P700 oxidation-reduction (redox) state were monitored simultaneously after each tomato seedling had adapted to darkness for at least 30 min, as described in previous studies [37,39,40]. Briefly, each measured leaf was illuminated for 8 min at a light intensity of 391 μmol·photons·m^−2^·s^−1^ to measure slow Chl fluorescence induction kinetics, and rapid light response curves (RLCs) of Chl fluorescence were recorded with the light intensity increased every 30 s (46, 87, 146, 251, 406, 634, 967, and 1204 μmol·photons·m^−2^·s^−1^). The following Chl fluorescence parameters were measured: the maximum photochemical quantum yield of PSII (Fv/Fm); the effective photochemical quantum yield of PSII (Y(II)); the quantum yield of non-regulated energy dissipation of PSII (Y(NO)); the quantum yield of regulated energy dissipation of PSII (Y(NPQ)). The following P700 redox parameters were measured: the maximum oxidation level of P700 (Pm); the photochemical quantum yield of PSI (Y(I)); the quantum yield of non-photochemical energy dissipation due to donor side limitation (Y(ND)); the quantum yield of non-photochemical energy dissipation due to acceptor side limitation (Y(NA)); the electron transport rate through PSI (ETR(I)); the electron transport rate through PSII (ETR(II)). The rate of cyclic electron flow (CEF) around PSI was calculated as the difference between ETR(I) and ETR(II) (ETR(I)-ETR(II)) [41,42]. The transient increase in Chl fluorescence after the actinic light (630 μmol photons m^–2^·s^–1^) was turned off was recorded as described in previous studies. Both slow Chl fluorescence induction kinetics and RLCs of Chl fluorescence images were recording using a MAXI Version Imaging PAM (Heinz Walz, Effeltrich, Germany) after each tomato seedling had adapted to darkness for at least 30 min. The light intensity was increased every 30 s (33, 102, 211, 359, 546, 653, 771, 897, 1022, 1177, and 1354 μmol·photons·m^−2^·s^−1^).

### 2.7. Transcriptomic Analysis

Frozen tomato leaf samples were ground in liquid nitrogen. Total RNA was extracted using the TRIzol reagent, according to the manufacturer’s instructions (Invitrogen, Waltham, MA, USA). The RNA was sequenced using the HiSeq 2500 sequencing system (Illumina, San Diego, CA, USA) at OeBiotech Co., Ltd. (Shanghai, China). The mRNA expression levels were calculated as fragments per kilobase per million mapped fragments (FPKM). The differentially expressed genes (DEGs) were identified according to the following criteria: absolute log2 fold change (FC) ≥ 2 and *p*-value < 0.05. Analyses based on the Kyoto Encyclopedia of Genes and Genomes (KEGG) and Gene Ontology (GO) annotation of DEGs were performed. Advanced heatmaps were plotted using the OmicStudio tools available at https://www.omicstudio.cn (accessed on 15 March 2022). Three biological replicates were used for each treatment.

### 2.8. Proteomic Analysis

Frozen tomato leaf samples were ground in liquid nitrogen. Total proteins were extracted using a phenol extraction method [43]. The concentration of total proteins was determined using a bicinchoninic acid (BCA) assay. Proteins were separated using 12% sodium dodecyl sulfate-polyacrylamide gel electrophoresis (SDS-PAGE). The trypsin-digested peptides were desalted using a C18-Reverse-Phase SPE Column (1100 Series HPLC System, Agilent, Santa Clara, CA, USA). Proteins were identified by liquid chromatography tandem mass spectrometry (LC-MS/MS) using a Q Exactive mass spectrometer (Thermo Fisher Scientific, Waltham, MA, USA) after chromatographic separation at Shanghai Lu-Ming Biotech Co., Ltd. (Shanghai, China). The LC-MS/MS raw data were imported into Spectronaut Pulsar (Biognosys, Schlieren, Switzerland) for quantification analysis using the Andromeda search engine (http://coxdocs.org/doku.php?id=maxquant:andromeda:start (accessed on 26 May 2019)). Mass spectrum data were searched against the ITAG 4.0 tomato protein database (https://solgenomics.net/organism/solanum_lycopersicum/genome (accessed on 29 May 2019)). Unique proteins with at least two unique peptides and a false discovery rate (FDR) < 0.01 were used for data analysis. The differentially expressed proteins (DEPs) were identified according to the following criteria: absolute log2 FC ≥ 2 and *p*-value < 0.05. KEGG and GO annotation of DEPs were performed. Advanced heatmaps were plotted using the OmicStudio tools available at https://www.omicstudio.cn (accessed on 27 March 2022). Three biological replicates were used for each treatment.

### 2.9. Statistical Analysis

Statistical differences (*p*-value < 0.05, denoted as *) between treatments were determined according to the student’s *t*-test performed in SPSS version 22 (SPSS, Chicago, IL, USA). Data are presented as the mean ± standard deviation of at least three independent biological replicates. Graphs were created using Origin version 12.0 (Systat, Chicago, IL, USA).

## 3. Results

### 3.1. The Effect of Melatonin on Photosynthetic Gas Exchange and Chloroplast Integrity

**Figure 1:**
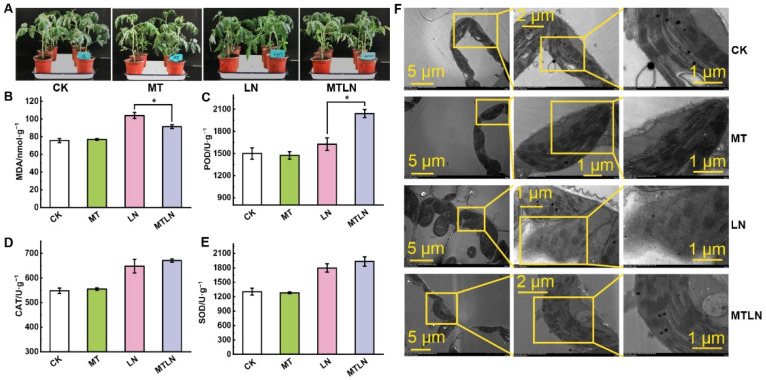
The effect of exogenous melatonin on the growth and antioxidant system of tomato plants subjected to low night temperature. Plant phenotype (**A**); malondialdehyde (MDA) content (**B**); peroxidase (POD) activity (**C**); catalase (CAT) activity (**D**); superoxide dismutase (SOD) activity (**E**); chloroplasts’ ultramicrostructures (**F**). Data are presented as the mean ± standard deviation of four independent biological replicates. * *p*-value < 0.05; student’s *t*-test.

**Figure 2:**
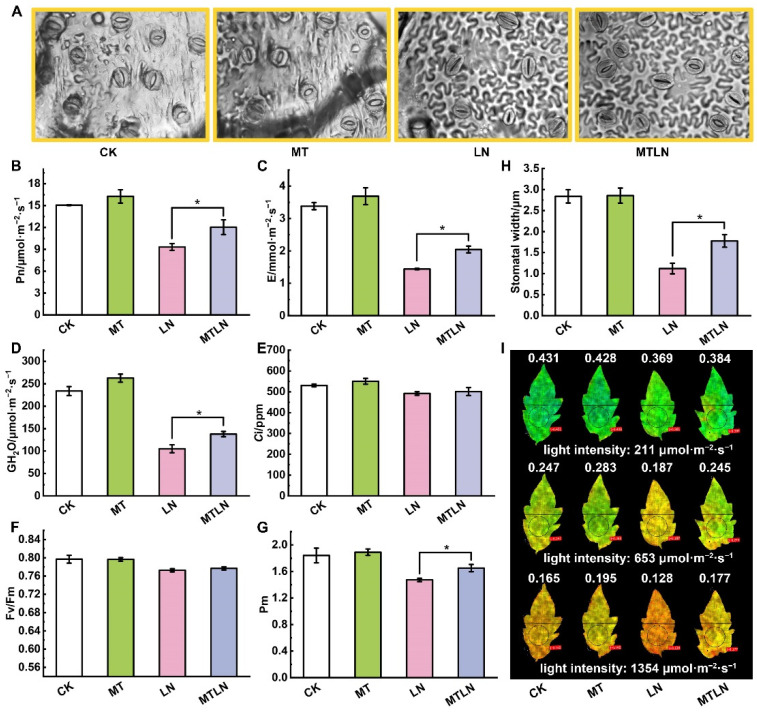
The effect of exogenous melatonin on photosynthetic gas exchange parameters and photosystem activity of tomato plants subjected to low night temperatures. Stomatal morphology (**A**); net photosynthetic rate (Pn) (**B**); transpiration rate (E) (**C**); stomatal conductance (GH_2_O) (**D**); intercellular carbon dioxide concentration (Ci) (**E**); maximum photochemical reaction efficiency of PSII (Fv/Fm) (**F**); maximum oxidation state of PSI (Pm) (**G**); stomatal width (**H**); chlorophyll fluorescence imaging of Y(II) (**I**). Data are presented as the mean ± standard deviation of four to six independent biological replicates. * *p*-value < 0.05; student’s *t*-test.

We observed no significant difference in the phenotypes between the control plants (CK) and plants treated with exogenous melatonin (MT) under a normal night temperature. Under a low night temperature, the untreated plants (LN) exhibited shorter stature and drooping leaves and a shrinkage phenotype, while the plants treated with melatonin (MTLN) exhibited normal phenotypes (Figure 1A). Under a low night temperature, the LN plants exhibited increased MDA content and decreased POD activity compared to the MTLN plants, although there was no significant difference in the CAT or POD activity between these groups (Figure 1B–E). The chloroplasts of the CK and MT plants were intact, with clearly crenellated thylakoid grana lamellae. The chloroplasts of the LN plants were damaged, with some chloroplasts exhibiting serious degradation or transportation to vacuoles. The chloroplasts of the MTLN plants were relatively intact, with visible grana lamellae, indicating that melatonin was able to alleviate chloroplast damage due to low temperature stress (Figure 1F). The low night temperature resulted in stomatal closure, with the melatonin pretreatment acting to increase stomatal apertures under low temperature stress (Figure 2A,H). The plants treated with melatonin exhibited higher Pn, E, and GH_2_O levels compared to the LN plants, although there was no difference in Ci between the groups, indicating that melatonin was able to increase the photosynthetic rate under low temperature stress (Figure 2B–E).

### 3.2. The Effect of Melatonin on Photosystem Activity and Light Energy Distribution

**Figure 3:**
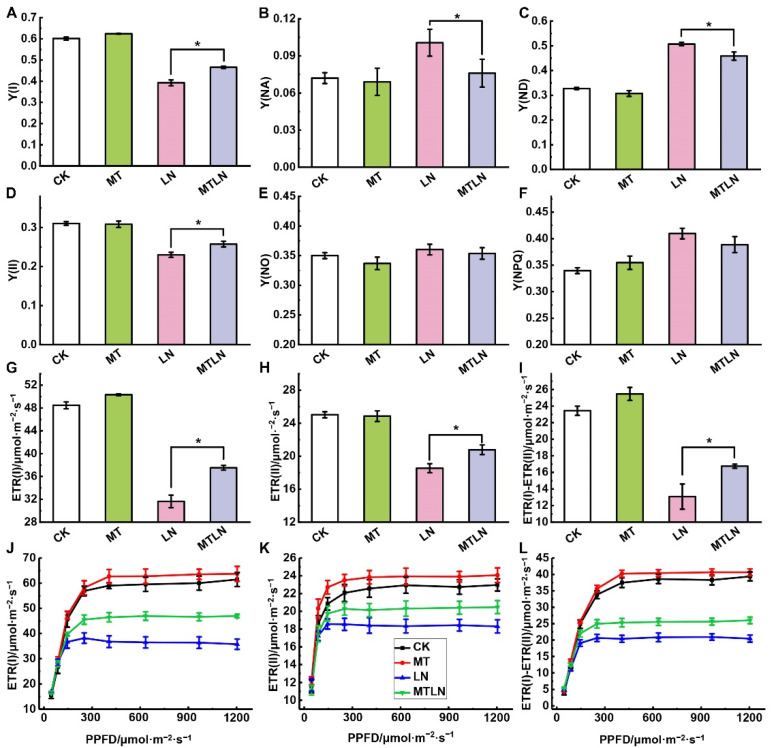
The effect of exogenous melatonin on light energy distribution and electron transfer dynamics of tomato plants subjected to low night temperature. The photochemical quantum yield of PSI (Y(I)) (**A**); the quantum yield of non-photochemical energy dissipation due to acceptor-side limitation (Y(NA)) (**B**); the quantum yield of non-photochemical energy dissipation due to donor-side limitation (Y(ND)) (**C**); the effective photochemical quantum yield of PSII (Y(II)) (**D**); the quantum yield of non-regulated energy dissipation of PSII (Y(NO)) (**E**); the quantum yield of regulated energy dissipation of PSII (Y(NPQ)) (**F**); the electron transport rate through PSI (ETR(I)) (**G**); the electron transport rate through PSII (ETR(II)) (**H**); the rate of cyclic electron flow (CEF) around PSI (ETR(I)-ETR(II)) (**I**) under steady light. The rapid light response curves of (ETR(I)) (**J**); the rapid light response curves of (ETR(II)) (**K**); the rapid light response curves of (ETR(I)-ETR(II)) (**L**). Data are presented as the mean ± standard deviation of four to six independent biological replicates. * *p*-value < 0.05; student’s *t*-test.

Exposure to low night temperature had little effect on the Fv/Fm but significantly reduced Pm. The Pm of the MTLN plants was significantly higher than the LN plants, indicating that melatonin was able to alleviate the photoinhibition of PSI due to low night temperature stress (Figure 2F,G). The plants treated with melatonin also exhibited higher Y(II) than the LN plants under different light intensities (Figure 2I). After 7 d of low night temperature exposure, the photochemical quantum yield, linear electron transport, and CEF around the PSI significantly decreased. As expected, the MTLN plants exhibited significantly higher Y(I), Y(II), ETR(I), ETR(II), and ETR(I)-ETR(II) than the LN plants (Figure 3A,D,G,H,I). According to the rapid light response curve of electron transfer, the difference was more obvious under relatively strong light (Figure 3J,K,L). Both Y(NO) and Y(NPQ) increased after exposure to low night temperature, although there was no significant difference between the treatments, suggesting that melatonin had little influence on PSII under low night temperature stress (Figure 3E,F). Both Y(NA) and Y(ND) levels were increased in the LN plants compared to the CK plants, and significantly increased compared to the MTLN plants, indicating that melatonin was able to protect PSI from damage by regulating the PSI donor- and acceptor-side restrictions (Figure 3B,C).

### 3.3. Melatonin-Mediated Photoprotection under Low Night Temperature Stress Involves SlPGR5/SlPGRL1-Dependent CEF 

**Figure 4:**
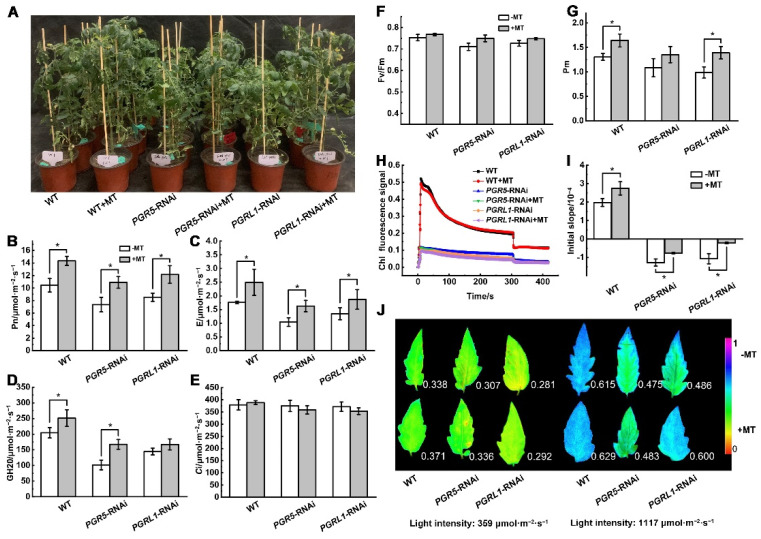
The effect of exogenous melatonin on photosynthetic gas exchange and chlorophyll fluorescence of tomato plants subjected to low night temperature. Plant phenotype (**A**); net photosynthetic rate (**B**); transpiration rate (**C**); stomatal conductance (**D**); intercellular carbon dioxide concentration (**E**); maximum photochemical reaction efficiency of PSⅡ (**F**); PSI maximum oxidation signal (**G**); chlorophyll fluorescence signal curve after turning the light off (**H**); the initial slope of the instantaneous increase of the signal after turning the light off (**I**); chlorophyll fluorescence imaging analysis of Y(NPQ) (**J**). Data are presented as the mean ± standard deviation of four to six independent biological replicates. * *p*-value < 0.05; student’s *t*-test.

To further validate the effect of an exogenous melatonin treatment on the regulation of the photosynthetic electron transfer rate, we used RNAi to genetically modify tomato plants to be defective in SlPGR5/SlPGRL1-dependent CEF. A phenotypic analysis showed that the plants (WT and RNAi) treated with melatonin were healthier and more robust than the untreated plants (Figure 4A). Furthermore, the plants treated with melatonin exhibited a higher net photosynthetic rate, transpiration rate, and stomatal conductance than untreated plants, although there was no significant difference in the intercellular CO_2_ concentration between groups, indicating that melatonin was able to alleviate the adverse effects of low night temperature stress on photosynthetic efficiency (Figure 4B–E). Under a low night temperature, the Pm of the melatonin-treated plants was significantly higher than in the untreated plants, although there was no significant difference in Fv/Fm between the groups, indicating that melatonin was able to alleviate the photoinhibition of PSI under low night temperature stress (Figure 4F,G). Compared to the WT plants, the chlorophyll fluorescence signal of the *PGRL1*-RNAi plants was extremely weak, and there was no instantaneous upward trend of the fluorescence signal after the actinic light was turned off. The initial slope of the signal increase was significantly higher in the melatonin-treated WT and RNAi plants compared to the untreated plants, indicating that melatonin improved the CEF rate under low night temperature stress (Figure 4H,I). Chlorophyll fluorescence imaging showed that Y(NPQ) was lower under low light and increased rapidly under a high light intensity, and that the plants treated with melatonin showed stronger NPQ ability under a high light intensity (Figure 4J).

### 3.4. The Effect of Melatonin on Gene Expression of Tomato Leaves under Low Night Temperature Stress

**Figure 5:**
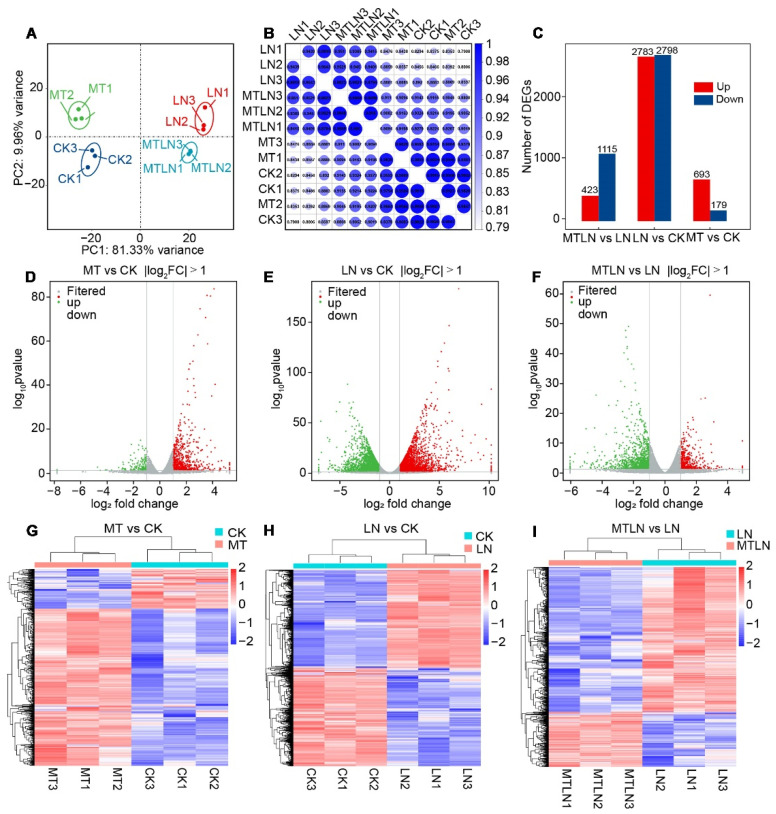
Analysis of differentially expressed genes (DEGs) of melatonin-treated and untreated tomato plants subjected to low night temperature. Principal component analysis (PCA) (**A**); heat diagram of correlation coefficient (**B**); the number of DEGs (**C**); volcano plot of DEGs between MT and CK (**D**); volcano plot of DEGs between LN and CK (**E**); volcano plot of DEGs between MTLN and LN (**F**); cluster heat map of DEGs between MT and CK (**G**); cluster heat map of DEGs between LN and CK (**H**); cluster heat map of DEGs between MTLN and LN (**I**).

**Figure 6:**
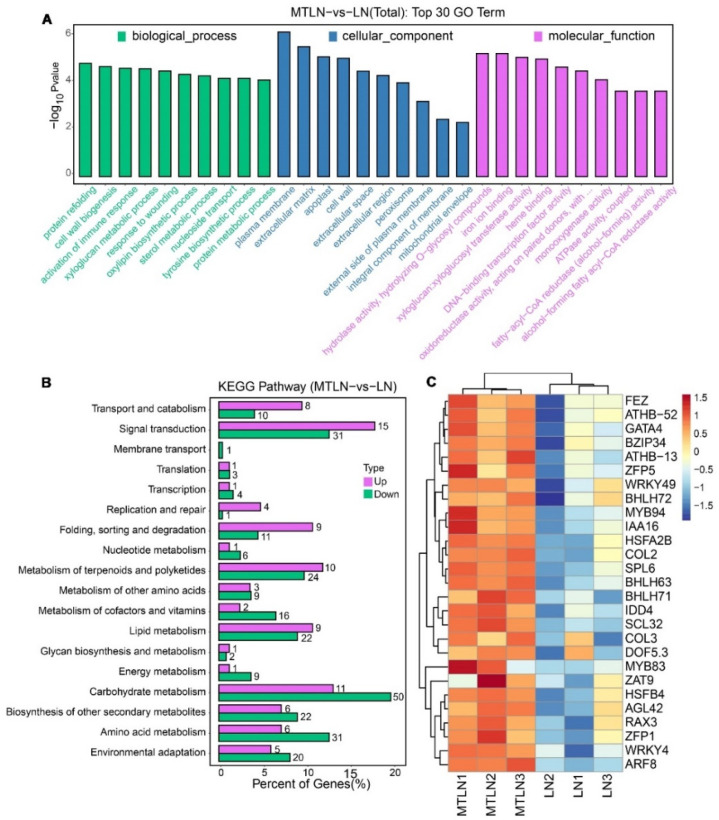
Go and KEGG enrichment analyses of melatonin in the regulation of DEGs in tomato leaves at low night temperatures. Analysis of enriched GO terms (**A**), KEGG metabolic pathways (**B**), and selected transcription factors upregulated by melatonin at low night temperature (**C**).

Transcriptomic sequencing was performed to study the effect of melatonin on transcriptional remodeling. A principal component analysis (PCA) showed that each group of samples was clustered together in a distinct quadrant, indicative of high intragroup repeatability (Figure 5A). The correlation coefficients of the sequenced samples for each treatment were all above 0.93, indicative of highly similar gene expression patterns within each treatment group (Figure 5B). Between the CK and LN plants, 2783 genes were up-regulated, and 2798 genes were down-regulated (Figure 5C). Between the CK and MT plants, 693 genes were up-regulated, and 179 genes were down-regulated (Figure 5C). Between the LN and MTLN plants, 423 genes were up-regulated, and 115 genes were down-regulated (Figure 5C). Volcano mapping was performed to visually assess the overall distribution of DEGs in the different comparison groups (Figure 5D–F), and cluster heat mapping was also performed for an analysis of the DEGs (Figure 5G–I). Here, we are primarily interested in comparing the DEGs between the MTLN and LN plants. Between these two groups, the GO functional annotation terms were enriched mainly in protein folding, cell wall synthesis, activated immune response, glucan metabolism, response injury, sterol metabolism, DNA-binding transcription factor activity, redox enzyme activity, and ATP synthase activity, among others (Figure 6A). Additionally, the KEGG metabolic pathways were primarily enriched in carbohydrate metabolism, terpenoid polyketone metabolism, genetic information processing, signal transduction, amino acid metabolism, lipid metabolism, and environmental adaptation, among others (Figure 6B). Finally, several genes encoding transcription factors (TFs) were up-regulated between these two groups, including the NAC domain transcription factors FEZ, Homeobox-leucine zipper protein ATHB-52, Basic leucine zipper 34 (BZIP34), Zinc finger protein of ZAT9, WRKY transcription factor 49, Heat stress transcription factor A-2b (HSFA2B), and Dof zinc finger protein DOF5.3, among others, suggesting that these TFs are involved in melatonin-induced resistance to low night temperature stress in tomato plants (Figure 6C and Table S1).

### 3.5. The Effect of Melatonin on Protein Expression of Tomato Leaves under Low Night Temperature Stress

**Figure 7:**
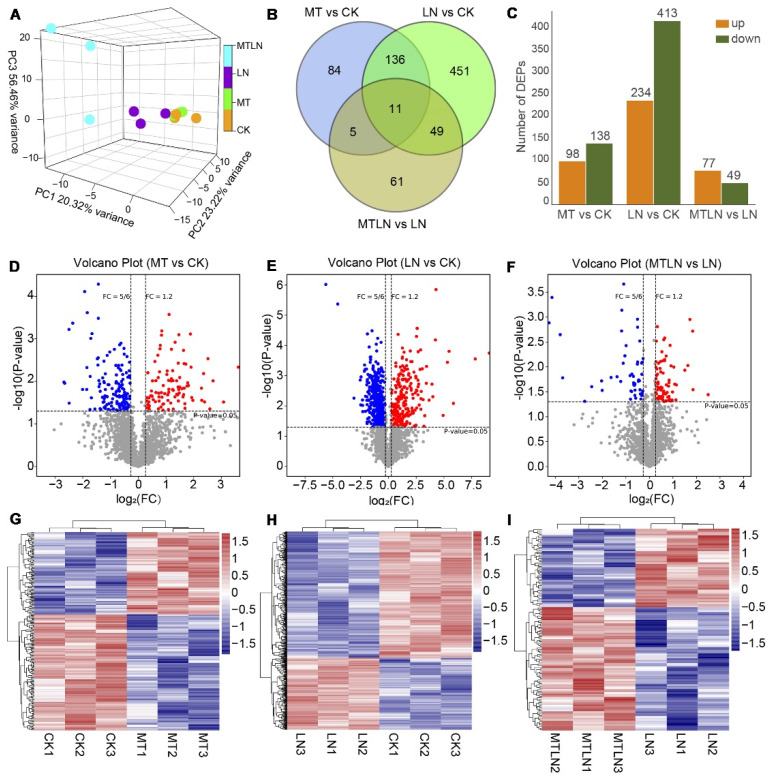
Analysis of differentially expressed proteins (DEPs) of melatonin-treated and untreated tomato plants subjected to low night temperature. Principal component analysis (PCA) (**A**); the number of DEPs (**B**); Venn diagram of DEPs (**C**); volcano plot of DEPs between MT and CK (**D**); volcano plot of DEPs between LN and CK (**E**); volcano plot of DEPs between MTLN and LN (**F**); cluster heat map of DEPs between MT and CK (**G**); cluster heat map of DEPs between LN and CK (**H**); cluster heat map of DEPs between MTLN and LN (**I**).

**Figure 8:**
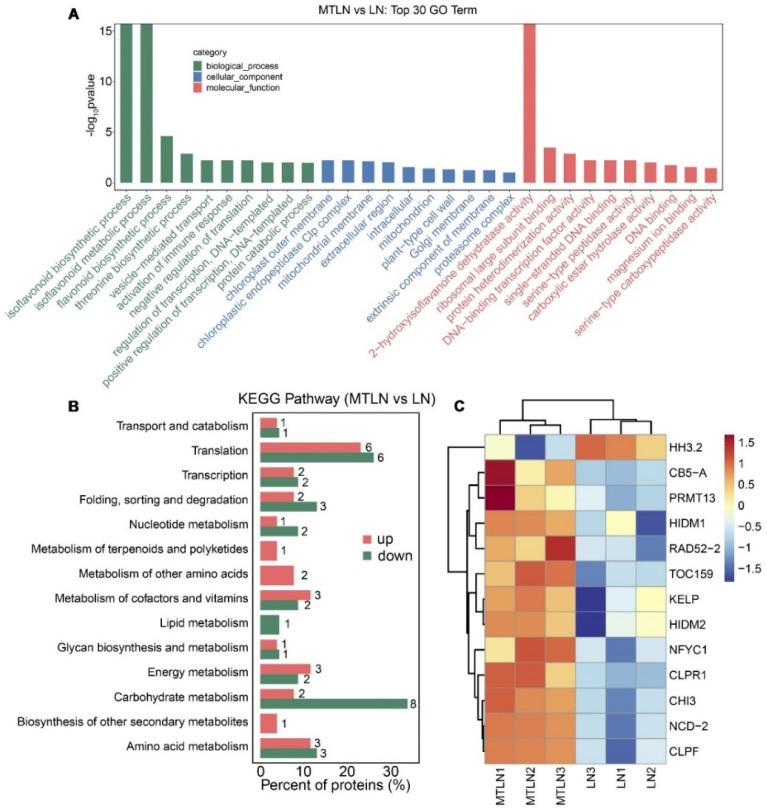
Go and KEGG enrichment analysis of melatonin in the regulation of DEPs in tomato leaves at low night temperatures. Analysis of enriched GO terms (**A**), KEGG metabolic pathways (**B**), and selected DEPs between LN and MTLN plants (**C**).

A proteomic analysis was performed to study the effect of melatonin on translational remodeling. The PCA showed that each group of samples was clustered together in a distinct quadrant, indicative of high intragroup repeatability (Figure 7A). A Venn diagram was created to visually assess the number of DEPs shared between the groups, indicating that 16 proteins were regulated by melatonin at normal and low temperatures and 120 proteins were regulated by melatonin specifically at low night temperatures (Figure 7B). Between CK and LN groups, 234 proteins were up-regulated, and 413 proteins were down-regulated (Figure 7C). Between the CK and MT groups, 98 proteins were up-regulated, and 138 proteins were down-regulated (Figure 7C). Between the LN and MTLN groups, 77 proteins were up-regulated, and 49 proteins were down-regulated (Figure 7C). Volcano mapping was performed to visually assess the overall distribution of DEPs in different comparison groups (Figure 7D–F), and cluster heat mapping was also performed for an analysis of the DEPs (Figure 7G–I). Here, we are primarily interested in comparing DEPs between the LN and MTLN plants. Between these two groups, the GO functional annotation terms were enriched mainly in the biosynthetic and metabolic processes of isoflavonoids and flavonoids, immune response, the chloroplast outer membrane, chloroplastic endopeptidase Clp complex, and DNA-binding transcription factor activity, among others (Figure 8A). Additionally, the KEGG metabolic pathways were primarily enriched in carbohydrate and energy metabolism, translation, the metabolism of amino acids, and the metabolism of cofactors and vitamins, among others (Figure 8B). Finally, several DEPs appeared to be regulated by melatonin specifically at low night temperatures, including transcriptional regulator histone H3.2 (HH3.2), nitronate monooxygenase (NCD2), nuclear transcription factor Y subunit C-1 (NFYC1), RNA polymerase II transcriptional coactivator (KELP), probable histone-arginine methyltransferase 13 (PRMT13), flavonoid metabolism-associated proteins 2-hydroxyisoflavanone dehydratase (HIDM1 and HIDM2), probable chalcone flavonone isomerase 3 (CHI3), chloroplast quality maintenance-related protein cytochrome b5 isoform A (CB5A), Clp protease adapter protein F (ClpF), and the translocase of chloroplast 159 (TOC159) (Figure 8C and Table S2).

## 4. Discussion

Melatonin has strong neuroendocrine-immunomodulatory activity, capable of regulating circadian rhythms and delaying aging, which has been widely used as a medicine in non-photosynthetic systems, such as humans and animals [2]. Melatonin can directly scavenge and detoxify free radicals, thus protecting plants from oxidative stress damage [2,33]. Herein, we studied the effect of an exogenous melatonin application on the photosynthetic response of tomato plants under low night temperature stress. We found that pretreating the tomato plants with 100 μM of melatonin significantly increased their stomatal apertures, carbon dioxide fixation rate, photochemical quantum yield of PSI and PSII, and electron transport rate under low night temperature. This is consistent with previous studies showing that melatonin confers tolerance to a broad spectrum of stressors. In melons, the application of melatonin was able to alleviate decreased photosynthetic efficiency and oxidative damage under salt (NaCl) stress, acting to inhibit stomatal closure and improve light energy absorption and the electron transport rate [44]. In an apple variety, the long-term application of 100 μM of melatonin to soil was shown to increase the photosynthetic activity, chlorophyll content, and sugar content of leaves [19]. In tomato plants, a treatment with 100 μM of melatonin has been shown to improve acid rain tolerance by increasing photosynthesis and endogenous antioxidant activity, as well as improving drought tolerance, acting to improve the net photosynthetic rate, transpiration rate, stomatal conductance, PSII quantum yield, electron transport rate, and Fv/Fm [45,46].

The excessive absorption of light energy induces photoinhibition and can lead to the accumulation of ROS in chloroplasts, and this effect is exacerbated under stress conditions [47]. A previous study found that exposing tomato plants to chilling stress resulted in the reduced photochemical reaction efficiency of PSII, which was alleviated by the application of melatonin [48]. While we found that melatonin application had little effect on the Fv/Fm under low temperature stress in this study, the photoinhibition of PSI was alleviated, thus reducing damage to chloroplasts. Studies in Arabidopsis, sunflowers, and rice have found that the PSI is extremely sensitive to light fluctuations and low temperatures [49,50,51]. The accumulation of electrons in the PSI leads to excessive ROS production, resulting in PSI inactivation [52]. The oxidation of P700 is a robust photoprotective mechanism that acts to suppress electron accumulation in the PSI and alleviate photoinhibition. Shorter-term regulatory mechanisms include nonphotochemical quenching (NPQ) and CEF, which have evolved to balance light-dependent photosynthetic processes [53]. What is noteworthy is that the regulation of both the Y(ND) and Y(NA) balance by CEF enables photoprotection [54].

Furthermore, SlPGR5/SlPGRL1-dependent CEF can effectively protect the PSI by alleviating pressure at the PSI acceptor side. It appears that melatonin has the ability to alleviate the photoinhibition caused by low temperature stress by regulating the limitations of the PSI at both the donor- and acceptor-side, and this photoprotective regulation is closely related to CEF. To characterize the relationship between melatonin application and SlPGR5/SlPGRL1-dependent CEF, *SlPGR5*- and *SlPGRL1*-RNAi plants were used in this study. We found both the WT and *SlPGR5*- and *SlPGRL1*-RNAi plants treated with melatonin grew better than the untreated plants when exposed to low night temperatures. Research shows that H_2_O_2_ (a byproduct of oxidative stress) can activate CEF either directly, through the modulation of key enzymes, or indirectly, though the modulation of photosynthetic processes; on the other hand, CEF-induced P700 oxidation has the ability to suppress ROS production in the PSI [55,56,57]. As a strong antioxidant, melatonin may relieve the oxidative damage of the photosynthetic machinery in both WT and SlPGR5/SlPGRL1-deficient tomato plants. However, the precise mechanism by which melatonin may exert these effects requires further study.

Although the multifunctional roles of melatonin in plants have been the subject of considerable study, integrated transcriptomic and proteomic profiling is lacking for many species and conditions. In Arabidopsis, a treatment with 100 pM of melatonin resulted in the identification of 81 DEGs, while a treatment with 1 mM of melatonin resulted in the identification of 1308 DEGs, many of which are involved in stress responses [58]. In salt-stressed rapeseed, a treatment with melatonin resulted in the identification of DEGs involved in phytohormone synthesis, signal transduction, and secondary metabolism [59]. In drought-stressed maize treated with melatonin, an iTRAQ-based proteomic analysis found an abundance of DEPs related to photosynthetic bio-sequestration, photosynthesis, amino acid biosynthesis, and secondary metabolite biosynthesis [60]. Furthermore, under osmotic stress, melatonin treatment has been found to significantly up-regulate the expression of glycolytic proteins, the autophagic response, and the protease- and ubiquitin-mediated protein degradation system in wheat [61]. In this study, the melatonin treatment induced the expression of a large number of genes and proteins related to metabolism and chloroplast integrity. The identified DEGs and DEPs should prove to be promising molecular targets for the continued characterization of the signaling pathways and downstream regulatory networks related to melatonin, and the molecular mechanism by which melatonin improves the photosynthetic efficiency of tomato plants under low temperature stress.

## 5. Conclusions

In order to verify the hypothesis previously proposed, we employed an integrated physiological, transcriptomic, and proteomic approach to study the effect of exogenous melatonin application on the resistance of tomato plants to low night temperatures. We found that the melatonin treatment significantly improved the carbon dioxide assimilation capacity and the photochemical reaction efficiency of low night temperature-stressed tomato plants. These effects could be attributed to the ability of melatonin to strengthen the photoprotection induced by SlPGR5/SlPGRL1-dependent CEF. Finally, melatonin application induces the expression of genes and proteins related to secondary metabolism, photosynthesis, and chloroplast integrity. Taken together, the melatonin-induced photosynthetic response and the changes in genes and proteins’ expression work together to improve the resistance of tomato plants to low night temperatures.

## Data Availability

The data that support the findings of this study are openly available in the National Center for Biotechnology Information (NCBI) SRA database under the BioProject ID: PRJNA875425 and Integrated Proteome Resources (iProX) database under dataset identifier IPX0004965000/PXD036438.

## Supplementary Materials

The following supporting information can be downloaded at: https://www.mdpi.com/article/10.3390/antiox11102060/s1, Table S1: Go and KEGG enrichment analysis of DEGs; Table S2: Go and KEGG enrichment analysis of DEPs.

## List of Abbreviations

reactive oxygen species (ROS), reactive nitrogen species (RNS), melatonin-2-hydroxylase (M2H), melatonin-3-hydroxylase (M3H), plant melatonin receptors (PMTRs), RNA interference (RNAi), *proton gradient regulation 5* (*PGR5*), *PGR5-like photosynthetic phenotype 1* (*PGRL1*), cyclic electron flow (CEF), phosphate-buffered saline (PBS), malondialdehyde (MDA), enzyme-linked immunosorbent assay (ELISA), superoxide dismutase (SOD), catalase (CAT), peroxidase (POD), transmission electron microscope (TEM), carbon dioxide (CO_2_), net photosynthetic rate (Pn), stomatal conductance (GH_2_O), intercellular CO_2_ concentration (Ci), transpiration rate (E), Chlorophyll (Chl), rapid light response curves (RLCs), differentially expressed genes (DEGs), Kyoto Encyclopedia of Genes and Genomes (KEGG), Gene Ontology (GO), differentially expressed proteins (DEPs), fold change (FC), bicinchoninic acid (BCA), sodium dodecyl sulfate-polyacrylamide gel electrophoresis (SDS-PAGE), and false discovery rate (FDR).
